# Supplementary material for: Resolving the full spectrum of human genome variation using Linked-Reads
Source: Genome Res. 2019 Apr;29(4):635–45. doi: 10.1101/gr.234443.118 (PMC6442396; doi:10.1101/gr.234443.118)
Supplement: Supplemental Material [file supp_gr.234443.118_Supplemental_longranger-2.2.zip › longranger-2.2.tar.bz2!/lr2.2-public/lib/stan/cmdstan/stan/doc/api/html/math_2matrix_2stan__print_8hpp_source.html]

Stan: src/stan/math/matrix/stan\_print.hpp Source File


|  |  |
| --- | --- |
|  | Stan  2.5.0  probability, sampling & optimization |


- Main Page
- Related Pages
- Namespaces
- Classes
- Files

- File List
- File Members

All Classes Namespaces Files Functions Variables Typedefs Enumerations Enumerator Friends Macros Pages

- src
- stan
- math
- matrix

stan\_print.hpp

Go to the documentation of this file.

1 #ifndef STAN\_\_MATH\_\_MATRIX\_\_STAN\_PRINT\_HPP

2 #define STAN\_\_MATH\_\_MATRIX\_\_STAN\_PRINT\_HPP

3

4 #include <vector>

5 #include <stan/math/matrix/Eigen.hpp>

6

7 namespace stan {

8  namespace math {

9  // prints used in generator for print() statements in modeling language

10

11  template <typename T>

12  void stan\_print(std::ostream\* o, const T& x) {

13  \*o << x;

14  }

15

16  template <typename T>

17  void stan\_print(std::ostream\* o, const std::vector<T>& x) {

18  \*o << '[';

19  for (int i = 0; i < x.size(); ++i) {

20  if (i > 0) \*o << ',';

21  stan\_print(o,x[i]);

22  }

23  \*o << ']';

24  }

25

26  template <typename T>

27  void stan\_print(std::ostream\* o, const Eigen::Matrix<T,Eigen::Dynamic,1>& x) {

28  \*o << '[';

29  for (int i = 0; i < x.size(); ++i) {

30  if (i > 0) \*o << ',';

31  stan\_print(o,x(i));

32  }

33  \*o << ']';

34  }

35

36  template <typename T>

37  void stan\_print(std::ostream\* o, const Eigen::Matrix<T,1,Eigen::Dynamic>& x) {

38  \*o << '[';

39  for (int i = 0; i < x.size(); ++i) {

40  if (i > 0) \*o << ',';

41  stan\_print(o,x(i));

42  }

43  \*o << ']';

44  }

45

46  template <typename T>

47  void stan\_print(std::ostream\* o,

48  const Eigen::Matrix<T,Eigen::Dynamic,Eigen::Dynamic>& x) {

49  \*o << '[';

50  for (int i = 0; i < x.rows(); ++i) {

51  if (i > 0) \*o << ',';

52  \*o << '[';

53  for (int j = 0; j < x.row(i).size(); ++j) {

54  if (j > 0) \*o << ',';

55  stan\_print(o,x.row(i)(j));

56  }

57  \*o << ']';

58  }

59  \*o << ']';

60  }

61

62  }

63 }

64 #endif

Eigen.hpp

stan::math::stan\_print

void stan\_print(std::ostream \*o, const T &x)

**Definition:** stan\_print.hpp:12


---

[ Stan Home Page ]

*© 2011–2014,
Stan Development Team.*
